# An Arrangement for Municipal Ownership of Slaughter-houses

**Published:** 1899-04

**Authors:** Leonard Pearson

**Affiliations:** Dean, University of Pennsylvania, Veterinary Department, Philadelphia, Pa.


### THE JOURNAL

OF

### COMPARATIVE MEDICINE AND VETERINARY ARCHIVES.

VOL. XX. *APRIL, 1899.* No. 4.

### AN ARRANGEMENT FOR MUNICIPAL OWNERSHIP OF SLAUGHTER-HOUSES.

BY LEONARD PEARSON, B.S., V.M.D.,

DEAN, UNIVERSITY OF PENNSYLVANIA, VETERINARY DEPARTMENT, PHILADELPHIA, PA.

THE American people are the greatest meat-eaters of the world, with the possible exception of the inhabitants of Australia. The beef-eating British consume nearly as much meat per capita as the Americans, and after these people we have the following, arranged in the order of the amount of meat consumed per capita and per annum: The inhabitants of Sweden, Norway, France, Germany, Belgium and Holland, Austria, Hungary, Russia, Spain, and Italy. The average annual consumption of meat in this country is about 115 pounds. The table that I have just given seems to support the assertion that the capacity of a people is indicated largely by the amount of flesh consumed.

Philadelphia uses about 125,000,000 pounds of meat every year. A great deal of this meat is prepared in Chicago and the other meat-packing centres of the West; but a large proportion of it is prepared in and about Philadelphia. Of the cattle that are slaughtered here, some are steers bred and fed in the West, others are Western-bred and Pennsylvania-fed, and others are young cattle reared in this and neighboring States; while still others are dairy-cows that have been fattened for the butcher, or they are worn out, thin, and ofttimes diseased cows that have become unprofitable for dairy purposes and are not suitable for feeding. In addition, the immediate vicinity supplies a considerable number of bulls and many veal calves.

Part of this meat of local origin is killed in the slaughter-houses of Philadelphia, and part of it in the small suburban slaughter-

[1] Read before the evening session of the Pennsylvania State Veterinary Medical Association, March, 1899.

houses or on the farms. The local slaughter-houses vary in capacity, from the little one in the yard back of the retail shop, where but one or two cattle can be handled a day, to the large one in West Philadelphia, with a capacity of several hundred. They vary as much in regard to sanitary arrangements, facilities for providing wholesome meat, and in cleanliness as they do in size. Some of the meat that is dressed outside of but near to Philadelphia is prepared in improvised slaughter-houses—a corner of the wagon-shed, or even of the barnyard. Many of the cattle killed in and about Philadelphia come from districts where tuberculosis is very prevalent. Instances have come to light where the owners of tuberculous cattle have sold them to the butcher after it was plainly evident that they were diseased, and, clearly, to avoid losing them by death. There are dealers who make a business of going about in dairy sections of this and other States to gather up all of the old, diseased, and worn-out cows that are able to walk. Such cattle are called "bolognas," and most of them are probably used in making the sausage that goes by that name.

Some of the smaller slaughter-houses in Philadelphia are of the most filthy and abominable character, and should be regarded as nuisances in the neighborhoods in which they exist. Their walls, floors, and fixtures are smeared and bespattered with blood, manure, and pieces of fat and meat that appear to have been accumulating since the buildings were erected, years ago. The live cattle are kept in compartments immediately adjoining the rooms in which animals are killed and meat is stored. The cattle are killed at irregular times, occasionally early in the morning, or at night, and the methods of dressing the carcass and preparing the meat for market are frequently of a dirty and unwholesome character.

For the purpose of inspecting the meat slaughtered in Philadelphia there are two "meat-detectives," who have associated with them two veterinarians, to be called in when the detectives find conditions that they consider suspicious. These detectives visit every day as many slaughter-houses as it is possible for them to reach, making a short visit at each one. If, during the time of their visit, they should happen to find diseased organs or diseased meat the veterinarian is called in for an expert opinion, and the meat is condemned, or not, according to his verdict. During the ninety-nine-one hundredths of the day when the meat-inspector is not present the slaughter-houses are not under inspection or control. It cannot be said, therefore, that there is a general system of meat-inspection in Philadelphia. The inspectors examine as much meat and as many cattle as it is possible for them to see, and are constantly employed. By their efforts a large quantity of dangerous meat is kept out of the market, and the health of the public is protected to an important degree, but the force employed by the city is utterly inadequate. I do not wish these remarks to be construed as a criticism of the city's meat-inspectors. It is rather a criticism of the city's meat-inspection system—a thing for which the inspectors are not responsible.

The need of an efficient and thorough system of meat-inspection is discussed by others at this meeting, so that I need only touch upon this portion of the general subject. Since the bacterial origin of many diseases has been demonstrated and the close relationship of some diseases of man to those of animals has been established, the importance of rational meat-inspection has been greatly emphasized. More than this, it is known that many parasites are transmitted to man through meat, and it is known that meat is composed of fragile compounds that change readily and render meat irritant or toxic unless it is handled in a cleanly, careful way, and is kept at a proper temperature. This need for meat-inspection is greatest in the portions of the country that have the largest proportion of diseased and worn-out animals that are unfit to become part of the people's food-supply. The need for meat-inspection is as great in the eastern part of the United States as it is in many parts of Europe, where it is carried out with the greatest degree of thoroughness and care.

The early meat-inspectors of Germany labored under the disadvantage that the meat-inspectors of Philadelphia are now forced to encounter. It was soon found, however, that these disadvantages were great enough to prevent meat-inspection from making the important contribution to the cause of public health that was expected of it, and, after many experiments and much thought, the plan was adopted by about 600 cities of causing the business of preparing meat-producing animals for food to unite in central establishments, usually the property of the municipality.

The system of municipal slaughter-houses is now so well established in Germany that every city that makes any pretence to progress is supplied with one of these important adjuncts to its sanitary system. Where the cities do not own slaughter-houses they regulate the business of slaughtering in private slaughter-houses by requiring these establishments to meet a certain standard, and they prescribe the time during which animals may be killed for food, so that the official inspectors may be on hand to examine every animal and carcass. Where the city owns the slaughter-house it requires all slaughtering carried on within its limits to be done in this one establishment. Thus, slaughter-houses are under the constant supervision of veterinarians, and all meat-producing animals are examined during life and again at the time they are killed and dressed.

Municipal slaughter-houses are in most cases model establishments. They contain all of the latest improvements in construction, and all of the approved modern appliances for the rapid, economical, and wholesome preparation and preservation of meat. They are in most cases supplied with smooth cement floors, suitably drained; they have brick or tiled walls, hot and cold water, arrangements for rapidly hoisting and moving animals and carcasses, and appropriate and adequate cold-storage facilities; they are plentifully illuminated and ventilated, and are kept in a condition of perfect cleanliness and altogether are as different from some of the small, close, dark, and ill-smelling little slaughter-houses of Philadelphia as a palace from a hovel.

This system of controlling the meat-supply is not confined to Germany alone, but is followed in nearly all parts of Europe, and by a number of cities in Great Britain. In this country the cities of New Orleans and Montgomery have also adopted the municipal abbatoir system, and with the most satisfactory results.

When carried out thoroughly and properly the control of the meat-supply gives important returns in many ways. It is, first of all, a great protection to the consumers of meat, and in the cities in which it has been established, helminthic diseases, meat-poisoning, and tuberculosis have been very materially restricted. Moreover, the meat that reaches the market is of a more attractive and nutritious character, and can be eaten with confidence and relish. This alone is a great gain. A general system of meat-inspection is also of the highest value in controlling the diseases of animals. The statistics as to the prevalence of disease made by meat-inspectors are the most complete and valuable that we possess. Another advantage of this practice is that it helps the honest butcher who wishes to furnish nothing but good meat to his customers, because it makes it unnecessary for him to compete with the man who sells carrion.

Objections to this plan come from two sources: from the owners of private slaughter-houses, who do not wish to be disturbed, and from people who believe that if all slaughtering were done in large municipal abbatoirs meat would be more expensive and, therefore, more difficult for the poor man to obtain. As to the first objection, it may be said that every advance interferes with earlier arrangements and upsets pre-existing conditions. The stage-coach proprietor and the canal-boatman objected to the building of railroads. All labor-saving machinery has been objected to by the people that it displaced; but reforms that improve general conditions and are good for the public cannot be permanently halted by the interests of individuals, and history shows that, in the end, the suffering that was prophesied from these changes has not come to pass. But many proprietors of slaughter-houses have at this time large interests and vested rights that must be respected. If the city of Philadelphia shall ever wish to establish a municipal abbatoir it will not be necessary for all butchers to move into this establishment at once. Those who maintain their private plants in conformity with certain regulations may be permitted to continue them for a stated period, while the smaller, filthier concerns that cannot be improved and are nuisances should be forced to discontinue as isolated establishments, and go into the large slaughter-house belonging to the city. For the conveniences provided a reasonable rental should be charged, and those who thus purchase the right to use the facilities of the municipal slaughter-house would be able to carry on their business under the best conditions—conditions that it would be absolutely impossible for most of them to provide at their own expense. They would be enabled to do their work in a proper, cleanly way; they would have the advantage of cold-storage facilities to a degree that the individual could not have; they could dispose of their condemned organs and carcasses to better advantage than now; they would have the advantage of easy access to the stock-yards, and would thus avoid the necessity of driving cattle through the streets, a practice that blocks the traffic, frightens people, and at times occasions serious accidents. I believe that if all the meat killed in Philadelphia were prepared in a large central slaughter-house under municipal control there would soon arise a strong demand for this meat. It would be preferred, and would make its own market. I see no other way by which the gradual destruction of our local slaughtering industry by the competition from the Western packers can be averted.

Now, as to the second objection to this plan, namely, the allegation that the cost of meat prepared in this way would be increased, and the poor man would have greater difficulty in providing meat for his family. This objection is old; it has been made and met in Germany as many times as there are public slaughter-houses, and has been discussed thoroughly by representatives of the meat industry, sanitarians, and others. In 1897 the director of the municipal slaughter-houses in Stockholm instituted an extensive inquiry for the purpose of obtaining information on the following points:

1. Whether it has been shown that the introduction of municipal slaughter-houses and obligatory inspection of meat brought into the city from outside has made meat more expensive and, in such case, to what extent has the price been increased.

2. Whether the municipal slaughter-house has been an earning institution.

3. As to the charges for killing and inspection.

Information on these points was received from 403 cities in Europe, and it was found:

1. That the price of meat had not become increased by the requirements as to the slaughtering and inspection.

2. That the public slaughter-houses were paying institutions.

3. That the price of meat depended upon the relation between supply and demand, and especially upon the price of cattle.

4. That the quality of the meat improved after the public slaughter-houses were opened, so that there was relatively a diminution in price.

5. That sometimes the price of meat went up immediately after the opening of the slaughter-houses, or that the butchers tried to increase the price, but very soon, as a result of competition, the price returned to its former level.

6. That in cities with public slaughter-houses and obligatory inspection the price of meat was not higher than in neighboring cities without these institutions.

7. That the freedom of occupation was increased rather than diminished after municipal slaughter-houses were erected, because every one who fulfilled the public requirements had the privilege to use the establishment. (Previously meat-producing animals could be slaughtered only by those who possessed the necessary premises and appliances.)

This extensive investigation has shown what economists would expect, but what there has been a great tendency to doubt. It has shown that by the species of coöperation that must be practised where large, fully equipped, and conveniently arranged municipal slaughter-houses are provided, meat can be prepared at a lower cost than where it is prepared in a multitude of small slaughter-houses.

